# Review of Dilaceration of Maxillary Central Incisor: A Mutidisciplinary Challenge

**DOI:** 10.5005/jp-journals-10005-1341

**Published:** 2016-04-22

**Authors:** Pawanjit Singh Walia, Ajit Kumar Rohilla, Shweta Choudhary, Ravneet Kaur

**Affiliations:** 1 Professor and Head, Department of Orthodontics, PDM Dental College and Research Institute, Bahadurgarh, Haryana, India; 2 Reader, Department of Orthodontics, PDM Dental College and Research Institute, Bahadurgarh, Haryana, India; 3 Reader, Department of Prosthodontics, PDM Dental College and Research Institute, Bahadurgarh, Haryana, India; 4 Senior Lecturer, Department of Orthodontics, PDM Dental College and Research Institute, Bahadurgarh, Haryana, India

**Keywords:** Dilaceration, Maxillary central incisor, Multidisci-plinary management, Trauma.

## Abstract

Traumatic injuries to primary dentition may interfere with the development of permanent dentition. Among the many malformations, dilaceration is particularly important to the clinician. Management of dilacerated maxillary central incisor requires a multidisciplinary approach.

The main purpose of this review is to present the etiological factors, the mechanism, clinical features, radiographic features and treatment of dilaceration of the maxillary central incisors.

**How to cite this article:** Walia PS, Rohilla AK, Choudhary S, Kaur R. Review of Dilaceration of Maxillary Central Incisor: A Multidisciplinary Challenge. Int J Clin Pediatr Dent 2016;9(1):90-98.

## INTRODUCTION

The term dilaceration was first coined in 1848 by Tomes,^1^ who defined the phenomenon as the forcible separation of the cap of the developed dentin from the pulp in which the development of the dentin is still progressing. Later, it was defined as an angulation or deviation or sharp bend or curve in the linear relationship of the crown of a tooth to its root (Latin: dilacero = tear up).^2,3^ According to the glossary of dental terms,^4^ dilaceration is defined as the deformity of a tooth due to a disturbance between the unmineralized and mineralized portions of the developing tooth germ.

**Fig. 1:**
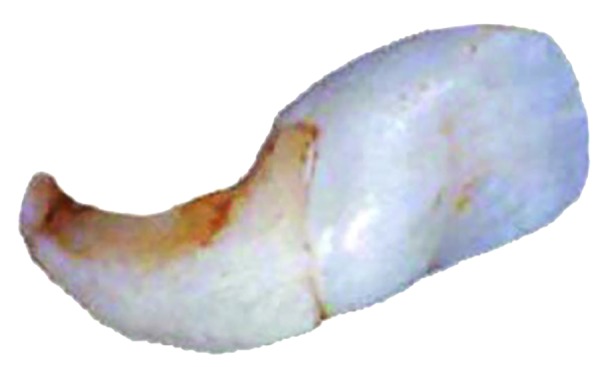
An extracted dilacerated maxillary central incisor tooth showing “classic” dilaceration

Andreasen et al, in 1971,^5,6^ defined dilaceration as the abrupt deviation of the long axis of the crown or root portion of the tooth, which is due to a traumatic nonaxial displacement of already formed hard tissue in relation to the developing soft tissue. The term vestibular root angulation (“sickle” incisor) is distinguished from dilaceration, as it denotes a curvature of the root resulting from a gradual change in the direction of root development without any evidence of abrupt displacement of the tooth germ during odontogenesis.^3,5,6^ Becker^7^ has described this condition as “classic” dilaceration (Fig. 1).

Stewart^8^ has likened tooth dilaceration to the hand of a traffic policeman, whereas Moreau^9^ used the term scorpion tooth for this condition. The criteria for recognizing root dilaceration vary in the literature. According to some authors, a tooth is considered to have a dilaceration toward mesial or distal direction if there is a 90° angle or greater along the axis of the tooth or root,^10,11^ whereas others defined dilaceration as a deviation from the normal axis of the tooth of 20° or more in the apical part of the root.^12^

## ETIOLOGY

The etiology of dilaceration is not fully understood and there is no consensus among researchers, although there are two prevailing explanations: The most widely accepted cause of dilaceration is acute mechanical injury to the primary predecessor tooth that leads to the dilaceration of the underlying developing succedaneous permanent tooth.

The calcified portion of the permanent tooth germ is displaced in such a way that the remainder of the noncalcified part of the permanent tooth germ forms an angle to it.^13-18^ Although the prevalence of traumatic injuries to the primary dentition ranges from 11 to 30%, the incidence of dilacerated permanent teeth is very low and disproportionate to the high prevalence of trauma. Hence, traumatic injuries to the primary dentition are unlikely to account for all cases of dilaceration and especially those of primary teeth themselves.^14,19^

In 1978, Stewart^8^ studied the phenomenon in 41 cases of dilacerated incisors and found that only in 22% (nine patients) of the cases, this was due to injury. Therefore, he concluded that the cause lay in the ectopic development of the tooth germ.

McNamara et al^18^ have reported that there are many studies which have found no history of trauma in cases of dilaceration. Also only a single maxillary central incisor presents dilaceration, whereas if injury was the only etiological factor, then adjacent teeth should be involved in the dilaceration more often. Therefore, it has been suggested that injury of a primary predecessor tooth is not the exclusive etiological factor of dilaceration.

The second explanation proposes an idiopathic developmental disturbance as the cause of dilacerations especially in cases that have no clear evidence of traumatic injury.^5,13,20,21^ Supporters of this theory maintain that an injury to a primary tooth sometimes leads to intrusion or avulsion, an event that normally occurs before the age of 4. At this age, the formation of the root of the succedaneous permanent tooth does not start. Therefore, injury is not the main etiological factor of dilaceration and this disorder is caused by ectopic tooth germ development.^5,20-23^ This theory is more acceptable because dilaceration is observed more frequently in posterior teeth, which are less susceptible to traumatic injury.^10^

Other possible contributing factors mentioned in the literature include the formation of scar tissue, developmental disorder in the primary tooth germ, facial clefting,^24^ advanced infection of root canals,^25^ ectopic tooth germ development and lack of space,^5,8,11^ the effect of anatomical structures, e.g., the cortical bone of the maxillary sinus, the mandibular canal and the nasal fossa, which may shift the epithelial diaphragm.^26^ Orotracheal intubation and laryngoscopy^27-29^ as well as the presence of cysts, tumors, odontogenic hamartoma,^8,27,30-32^ mechanical interference during eruption, such as an ankylotic primary tooth, the roots of which are nonresorbed,^29^ tooth transplantation,^33^ extraction of a primary tooth,^34^ and hereditary factors.^35-39^ Certain syndromes and developmental disorders have also been associated with root dilaceration, such as, Smith-Magenis syndrome,^38^ the hypermobility type of Ehlers-Danlos syndrome,^39^ the Axenfeld-Rieger syndrome,^40^ and congenital ichthyosis.^41^

## MECHANISM CAUSING DILACERATION

In early developmental stages, the permanent tooth germ of the maxillary incisor is situated palatally and superiorly to the apex of the primary incisor and gradually changes its path in a labial direction with its crown coming closer to the resorbing primary root. The fibrous connective tissue present between deciduous maxillary central incisor and succedaneous permanent maxillary incisor is <3 mm in thickness.^42,43^ Injury to the primary central incisor leads to various developmental disorders in the developing permanent tooth bud due to close anatomical relationships between the permanent tooth bud and the root of the primary central incisior.^42,44-46^ Dilaceration is one such disorder, the position of which will depend on the developmental stage of the tooth at the time of the injury.^6,7^

The impact force on primary incisor which is vertically directed is transferred in the direction of the longitudinal axis and it may be carried along the apex to the noncalci-fied or partially calcified tooth germ of the permanent successor.^14^

**Fig. 2:**
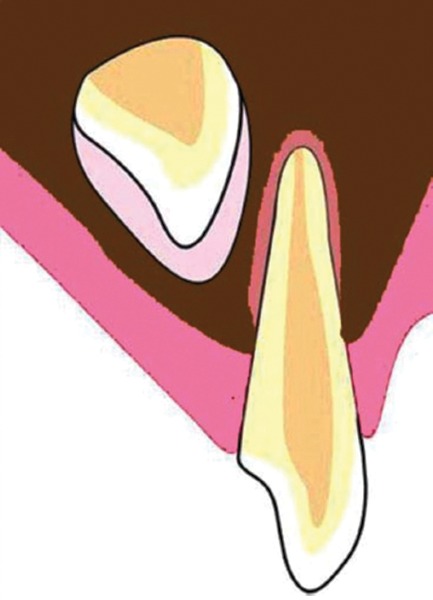
Close proximity of maxillary deciduous and permanent successor tooth germ at 2-3 years of age

**Fig. 3:**
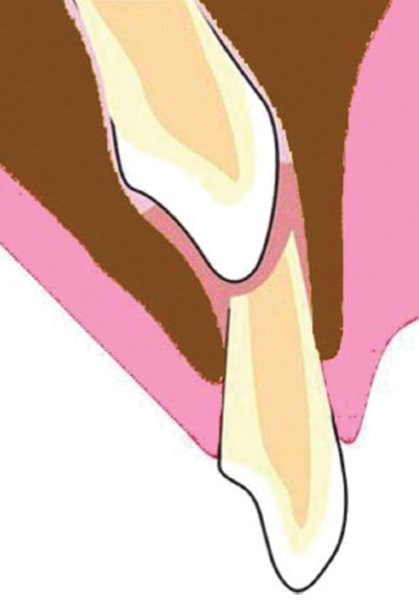
Close proximity of maxillary deciduous and permanent successor tooth germ at 4-5 years of age

**Fig. 4:**
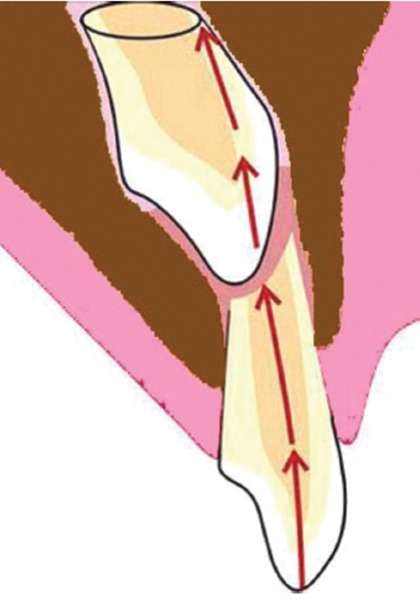
Vertically directed force through the deciduous incisor transmitted to the labial aspect of the mineralizing root of unerupted permanent incisor

If injury to the primary tooth occurs at the age of 2-3 years, buccal surface of the permanent maxillary incisor tooth would be affected as the tooth germ of the permanent maxillary incisor lies in a palatal position, above the apex of the primary incisor^44^ (Fig. 2). At the age of 4-5 years, the tooth germ of the permanent incisor shifts toward the labial direction, thus coming closer to the resorbing root of the primary tooth^45,47^ (Fig. 3). At this critical age, when the crown of the permanent tooth is in direct relationship with the resorbed root of the primary predecessor, if the child is injured, the impact force will be transferred along an imaginary oblique line that goes through the incisal edge of the permanent incisor and a point on the labial aspect of its newly formed root^7^ (Fig. 4). It is appraised that the direction of this force may be more significant than its magnitude. As the impact force is directly transferred to the cells of Hertwig’s epithelial root sheath, through the sharp end of the nonformed root of the permanent tooth, it is possible for serious damage to be caused despite the relatively mild forces involved. The resorbing apex of the primary incisor creates an impact point with the incisal edge of the crown of the permanent incisor and causes this crown to turn upward into its tooth follicle.^6,7,14,45^ As the permanent incisor root has not been fully developed at the moment of injury, the part of the root already formed will rotate along with the crown. However, further root development, following the injury, usually continues in the same direction it was following before the injury. This creates an unusual angle between the pre- and the posttraumatic parts of the tooth, which results in local curvature of the longitudinal axis of the permanent central incisor and causes dilaceration.^7^

**Fig. 5:**
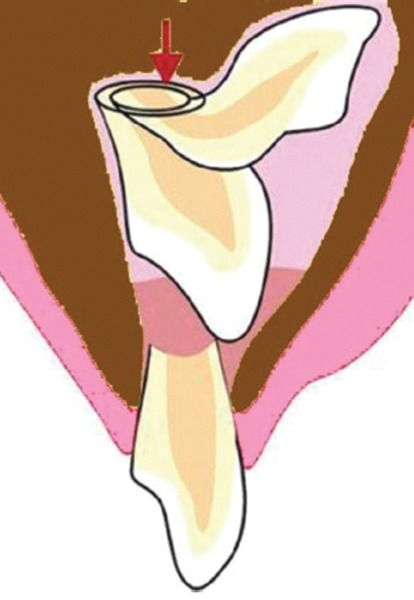
Progressive alteration in the direction of a dilacerated incisor during unequal root formation following traumatic injury. The position of Hertwig’s epithelial root sheath remains unaltered

As the injured Hertwig’s epithelial root sheath continues to produce dentin at the same rate as before the injury, the final root shape of the permanent maxillary central incisor will be formed in a continuous labial curve, until apex formation has been completed.^6,7^ Furthermore, as the Hertwig’s epithelial root sheath remains in its place within the alveolar process against the eruptive forces of the developing tooth and guides the orientation of root development, the crown of the permanent central incisor appears to be moving labially and upward for as long as this asymmetric calcification of the root continues^5-7^ (Fig. 5). Therefore, dilaceration of this classical type is an anomaly which is traumatic in origin and developmental in its final expression. This mechanism explains the typical appearance of dilacerated tooth with a relatively minor degree of trauma and high proportion of cases with no apparent history of trauma and no damage to the adjacent teeth. It also provides explanation for bilaterally affected cases, nonoccurrence among lateral incisor and absence of any association with supernumerary teeth, cyst and odontoma.^7^

## EPIDEMIOLOGY

Dilaceration may appear in both permanent and primary teeth but incidence in the latter is very low.^13,18,48^ While some studies report no gender preference for dilaceration,^49^ others report a male to female ratio of 1:6.^8,18^ In 2006, Malcic et al^11^ reported a prevalence rate of 1.3 or 0.53% for maxillary central incisors on the basis of periapical and panoramic radiographs respectively. Hamasha et al^10^ examined 4,655 teeth on periapical radiographs and found that 176 (3.78%) presented dilaceration. Maxillary central and lateral incisors had rates of 0.4 and 1.2% respectively.

Malcic et al^11^ reported that dilaceration is observed in the apical third of the roots of incisor, canines and premolars, while the middle third is more often affected in molars and finally, the cervical third in third molars. These authors also reported that premolars and maxillary anterior teeth present a higher total prevalence (4.6%) as compared with the rate affecting the corresponding region of the mandible (1.3%).

Dilaceration might occur anywhere along the length of the tooth, i.e., the crown, the cement-enamel junction, along the root or the root apex.^2,3,11,18^ Crown dilaceration of a permanent tooth constitutes 3% of all traumatic injuries to developing teeth and is habitually due to intrusion or avulsion of their primary predecessors, and it usually involves maxillary or mandibular central incisors.^5,6^ Maxillary incisors are more often involved than their mandibular counterparts.^17^ Approximately, 50% of teeth with crown dilaceration become impacted, with the remainder erupting normally or in a labiolingual direction.^6^ Crown dilac-eration of permanent maxillary incisors usually presents with palatal angulation, while permanent mandibular incisors usually present crown dilaceration with labial angulation.^5,50^ The clinical appearance of this deformity in succedaneous permanent tooth depends on the developing stage at which the injury occurred.^51^

Bilateral dilacerated teeth have been observed in the same patient,^52,53^ while the presence of dilacerated teeth in both the maxillary and mandibular dental arches in the same patient is quite a rare phenomenon.^36^

The most common type of dilaceration is that of a tooth root angulation combined with a reversal crown direction. The palatal aspect of the crown faces the labial side and the tooth is usually impacted.^8,18,54,55^ Becker^7^ has called this condition as classic dilaceration.

## CLINICAL FEATURES

The spectrum of clinical presentation for dilacerated tooth may vary from noneruption of the affected tooth, prolonged retention of the primary predecessor, apical fenestration of the buccal or labial cortical plate or it may be asymptomatic.^13,27,48,56^ The presence of dilaceration in an impacted maxillary central incisor may be diagnosed clinically through palpation at two sites. The first site lies high on the labial side of the alveolar ridge in the vestibular sulcus. The superior midline area is delineated by the prominence of the anterior nasal spine on either side of which a shallow depression can be felt. In cases of dilaceration of the permanent central incisors, when the palatal surface of the crown faces the labial surface, there is a noticeable bulge in place of the shallow depression. When the upper lip is pulled upward, the oral mucosa moves freely above the bulge, which indicates the outline of the cingulum area of the crown of the impacted dilacerated central incisor. If the palpation of this area is not performed meticulously, an important diagnosis may be missed.^7^

The second palpation area lies in the palate. With the abnormal position of the coronal portion of the tooth, such as when the crown has rotated upward and labially, the root continues to develop along a more palatally tilted axis. Therefore, at the final stages of incisor root formation when the apex is closed, the apex may be palpated in the palate as a small hard nodule. This feature is overlooked by most clinicians and is a more consistent finding than may be realized.^57^

## RADIOGRAPHIC FEATURES

The recognition and diagnosis of dilacerations are essential for any tooth that requires orthodontic treatment,^58^ root canal treatment^12^ or extraction.^59^ Dilaceration of a crown can be visually observed in the mouth (provided the tooth is not impacted); however, radiographic examination is required to diagnose dilaceration in the root.^10^

**Fig. 6:**
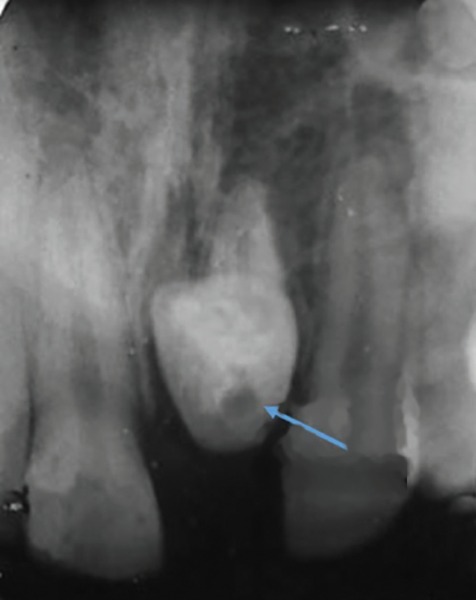
Periapical radiograph showing “Bulls Eye” phenomenon in a central incisor with dilacerated root

The direction of root dilaceration should be considered in two planes and they can be categorized as mesial, distal, labial/buccal or palatal/lingual. If the roots bend mesially or distally, the dilaceration is clearly apparent on a periapical radiograph. However, when the dilaceration is toward the labial/buccal or palatal/lingual, the central X-ray beam passes almost parallel to the deviating part of the root. The deviating root portion appears at the end of the nondeviating portion as a circular radiopaque region with a dark central radiolucent spot, which represents the apical foramen and is a part of the root canal as well. This radiographic image is known as a Bull’s Eye or a target (Fig. 6). The periodontal ligament around the deviating part of the root appears as a black region (radiolucent halo). The deviating portion of the root appears more radiopaque as compared with the rest of the root because the X-ray beam passes through a higher osseous density portion of the root.^21,60^

**Figs 7A to C:**
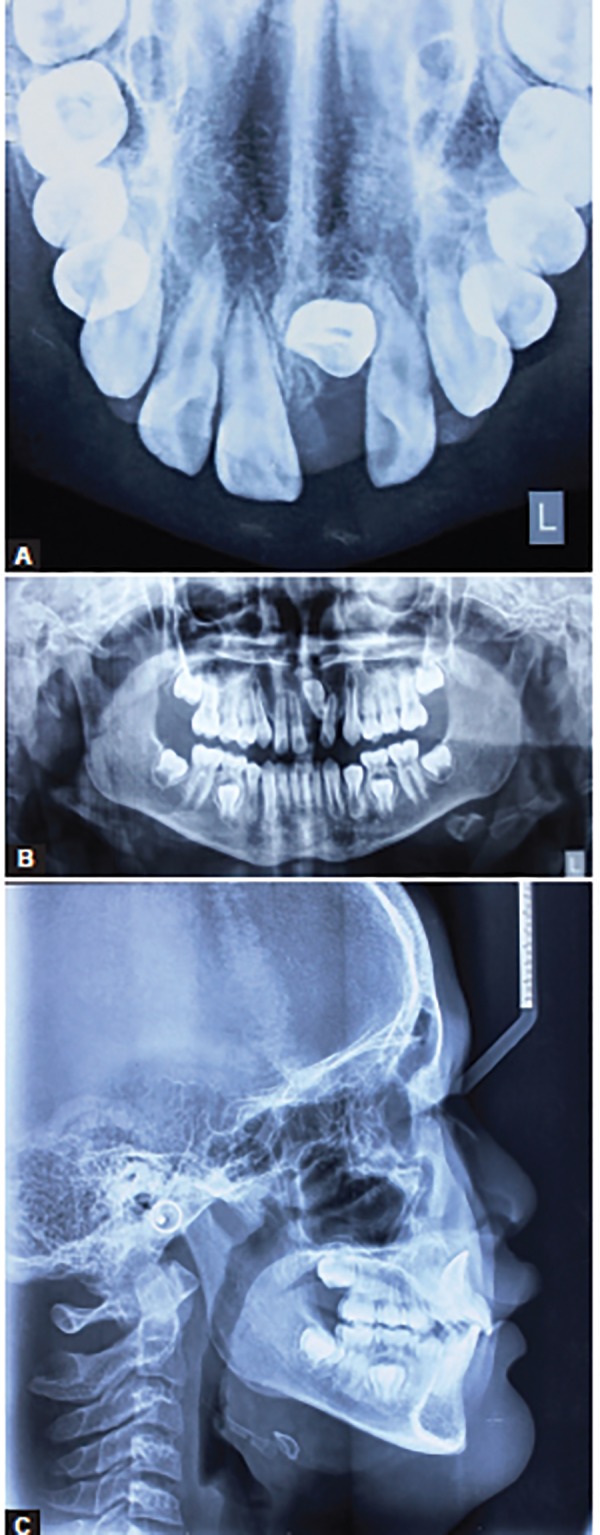
(A) Occlusal view of a patient with maxillary left permanent central incisor dilaceration, (B) panoramic radiographic view of a patient with maxillary left permanent central incisor dilaceration, and (C) lateral cephalogram of a patient with maxillary left permanent central incisor dilaceration

**Figs 8A and B:**
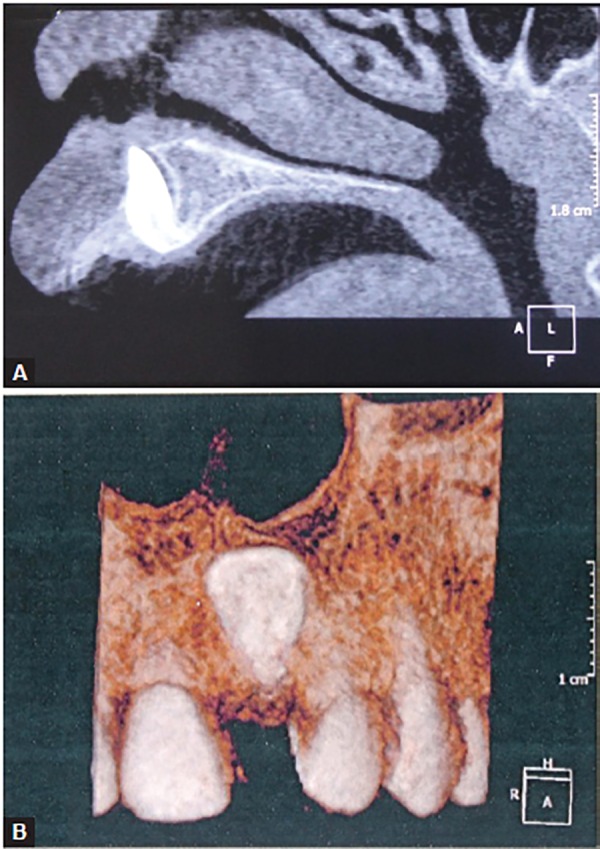
(A) Sagittal section of cone beam computed tomography image showing an impacted tooth root located palatally with a large part close to palatal cortical bone, and (B) three-dimensional frontal photographic reconstruction from CBCT image of a patient with maxillary left permanent central incisor dilaceration

Conventionally, radiographic diagnosis was based on two-dimensional (2D) radiographic images^61^ (Figs 7A to C). However, 2D radiographic images can be hindered by rotation, distortion and errors in head positioning, which cause inaccurate representations of anatomic landmarks and poor visualization of some anatomic structures.^62^ Cone beam computed tomography (CBCT) has been recently introduced in radiographic diagnosis of impacted teeth, since it provides multiple planes for accurately identifying three-dimensional (3D) landmarks of dental structures with submillimeter resolution.^63,64^ Cone beam computed tomography also provides various sections of the structure of interest, allowing clinicians to assess the exact positions of the apex and the crown, and the degrees of root formation and dilaceration^45^ (Figs 8A and B). The advantages of CBCT over conventional computed tomography or dental images include low radiation dose, low cost, excellent tissue contrast, elimination of blurring and overlapping of adjacent teeth and high spatial resolution.^64,65^ Therefore, the application of CBCT in the diagnosis and treatment of impacted dilacerated teeth has become increasingly indispensable.

## PROGNOSIS

The prognosis of aligning an impacted dilacerated tooth mainly depends on the following factors: (1) the position and direction of the impacted tooth, (2) the degree of root formation, (3) the degree of dilaceration, and (4) the availability of space for the impacted tooth.^66-69^ Machtei et al^70^ also include the condition of the periodontium. McNamara et al^18^ underline the decisive significance of the posttraumatic condition of the Hertwig’s epithelial root sheath for a successful therapeutic outcome, as the odontogenic epithelium plays a truly important role in root formation through the effect of its Hertwig’s epithelial root sheath. Continuing normal root development depends on the integrity of the Hertwig’s epithelial root sheath.^18,47^ A dilacerated tooth with an obtuse inclination angle, a lower position in relation to the alveolar crest combined with an incomplete root formation has a better prognosis for orthodontic traction.^67,69^ Chaushu et al^71^ reported that orthodontic surgical treatment of impacted central incisor is generally successful but relatively long and is significantly affected by the initial height of the impacted tooth.

## TREATMENT CONSIDERATIONS

To provide an opportunity for the noncalcified root to change direction and develop a proper spatial relationship with the already calcified formed crown, the treatment of dilacerated teeth should start early.^18^ Due to position of impacted dilacerated maxillary central incisor, the problem is usually recognized by the parents during the child’s mixed dentition period. Failure to treat in a timely manner may lead to delayed tooth eruption, midline shift, space occupation by adjacent teeth and alveolar crest height differences.^54^

Management of an impacted dilacerated permanent teeth includes two different treatment approaches: (1) surgical exposure with orthodontic traction^18,66-69^ or (2) extraction which may be followed by (a) space closure by mesializing the lateral incisor in place of the central incisor with subsequent prosthetic restoration,^72-74^ (b) surgical repositioning of the impacted central incisor,^54^ (c) autotransplantation of a premolar to the region^54,75^ and (d) restoration with an implant or a bridge after cessation of growth.^76,77^

Among these, orthodontically induced tooth eruption would be the first choice based on sound evidence of its benefits.^69,78-80^ It helps in maintaining tooth structure, provides bone stimulation and maintenance of alveolar bone width besides providing periodontal and esthetic benefits.^78^ This treatment although complex can be successfully managed by careful planning and by a multidisciplinary team including the pedodontist, orthodontist, maxillofacial surgeon, endodontist, and periodontist.^81^

Even after successful orthodontic treatment, esthetic periodontal surgery might be necessary if the final position of the gingival margin is not acceptable due to gingival recession and/or clinical crown lengthening.^60,69,70,82-85^ Orthodontists are often reluctant to proceed with aligning severely dilacerated teeth as treatment might fail due to complications such as ankylosis, loss of attachment, external root resorption and/or root exposure following orthodontic traction.^55,69,86^ In cases of root exposure, endodontic treatment and/or apicoectomy would be necessary.^55,68,69,85^

## CONCLUSION

Dilaceration of permanent teeth is a relatively rare phenomenon but when present they pose a multitude of diagnostic, prognostic, and management challenges.

In addition to routine clinical examination, radio-graphic examination is essential for diagnosing dilac-eration. To reach a definitive diagnosis and improve treatment planning, the role of latest imaging tools such as CBCT is indispensable. Treatment of dilacerated maxillary incisor impaction should start as early as possible and comprises surgical exposure followed by orthodontic traction and tooth alignment in the dental arch. For successful management a multidisciplinary approach and a high level of cooperation from the patient is required.
